# Construction Notes

**Published:** 1893-07-01

**Authors:** 


### CONSTRUCTION NOTES.

Croydon General Hospital Extension.—A new wing for the Croydon General Hospital has been designed by Mr. Henman. This wing will be thrown out at the back, so that the front portion of the hospital will remain untouched. By this there will be a gain of 24 additional beds. The cost for the same being estimated at between £9,000 and £10,000, it is proposed by the Committee to sell out £6,500 of Consols, and to appeal to the public for the remaining £2,500.

Lancaster New Infirmary.—It has become necessary to erect a new infirmary at Lancaster, as the present premises are inadequate and inconvenient. The site chosen is a portion of Springfield Park, and the building, according to the plans which have been prepared by Messrs. Paley, Austin, and Paley, will consist of one large ward, to contain 20 beds, and three small wards, to contain 10 beds each, with the necessary accommodation for administrative purposes, also a convalescent wing; and in addition there will be a children's ward to contain 10 beds, which was given, as before mentioned, by Mr. W. Smith, M.P. The total cost of the erection will be £21,700.

